# The Quack Must Go—First Gun of the Campaign

**Published:** 1888-09

**Authors:** 


### EDITORIAL DEPARTMENT.

F. E. DANIEL, M. D., EDITOR AND PUBLISHER.

THIS JOURNAL, by unanimous vote of the members, was made the Official Organ of the Austin District Medical Society.

COLLABORATORS:

*Dr. R. M. Swearingen, Austin.*
*Prof. B. E. Hadra, M. D., Galveston.*
*Prof. Geo. Cupples, M. D., San Antonio.*
*Dr. T. C. Osborn, Cleburne, Texas.*
*Dr E. J. Doering, Chicago.*
*Dr. E. J. Beall, Fort Worth.*
*Dr Odo Betz, Germany.*
*Dr. J. W. McLaughlin, Austin.*
*Dr. T. J. Tyner, Austin.*
*Prof. J. F. Y. Paine, M. D., Galveston.*
*Dr. R. H. L. Bibb, Mexico.*
*Dr. T. J. Bennett, Austin.*
*Dr. Bat Smith, Wharton.*
*Dr. E. Meierhof, New York.*
*Prof. W. B. Rogers, M. D., Memphis, Tenn.*

### THE QUACK MUST GO—FIRST GUN OF THE CAMPAIGN.

At the meeting of the Austin District Medical Society, which will be held in this city on the 20th instant, it is understood that a resolution will be introduced—and no doubt adopted—declaring that the physicians of Travis and adjacent counties represented in this Association, will not support with their ballot, and influence with their respective *clientelles*, any candidate for the Legislature who will not pledge himself to favor and advocate a bill before the Legislature to regulate the practice of medicine in this State.

This is a step which we would like to see followed by every organized body of physicians in Texas. The time for action and co-operation amongst the physicians has come. There is no use in talking, they must act, and act in concert. The sentiment is general, universal, we may say, amongst the educated members of the medical profession, that there should be some discrimination and distinction between them and the *pretenders.* They feel and claim, and with justice, that they, as well as the blessed people, should have some "protection"—protection in their *rights.* Those who have invested money, and devoted the best years of their lives to the acquisition of knowledge of a kind to make their services worth a money consideration, (to take a business view of the subject,) feel that there is injustice done them when a person, who has invested neither time, money nor labor in the pursuit of knowledge, and who has no knowledge of medicine, is permitted, under the law, to offer his services to do that which he is not qualified by education and training to do, and to charge for the same; thus coming in competition, and often successfully, with the trained and educated physicians. They feel, and we claim that the State, in justice to itself, to its citizens, and to the profession of medicine, should require a standard of qualification, and a test. The practice of medicine is a dangerous privilege. It should not be entrusted indiscriminately; its èxercise should be restricted to those who can give evidence of a fitness or qualification for it, no matter *how* or *where* obtained,—whether in a school called "Allopathic," or what not,—a test should be made, and those found unqualified,—totally ignorant of the fundamental principles of the science,—should be barred the privilege. The Homeopaths laugh at the efforts to procure a law to regulate the practice, and accuse the physicians of the State Association—the "regular profession"—of wanting protection for themselves. This is *true*, to some extent, and in the sense above set forth; but, are they so blind as not to see that, in a measure, it would protect the educated of their persuasion also, from bastard competition?

Such a measure as is advocated here, and which has been for years, advocated by the organized branch of the Texas profession, aims, primarily, at the *suppression of quackery*, the restriction of the privilege of practicing a dangerous pursuit to those who have been educated to it, and can practice it without jeopardizing the lives of the people; thus, it aims at the protection of the people's lives first, and incidentally only, affords a certain protection to the profession, in securing them against a species of competition which the State would not allow in any other calling or pursuit on earth; all other pursuits and privileges, of whatever nature, of equal or similar responsibility, are *licensed* by the State. An engineer on a railroad, is not permitted to take charge of a train load of passengers, until he has *stood an examination*, and shown himself competent, by education, to follow the calling he has adopted.

Every other effort to secure to passage of a medical act, has failed. The legislators, like the people, know no difference. A "doctor" to them, is a "doctor," and they put them all in one category. They say that efforts of the kind alluded to, are only a species of quarrel amongst the different *pathies*. It seems an Utopian task to undertake to "educate them" up to a point whence they can discriminate. The organized branches of medicine, in Texas, are driven to the course above indicated; they must go to the fountain head, and by the exercise of their influence, individually and collectively, and through their extensive family practice, *prevent the election* to the Legislature of the small-bore, "cimblin-headed statesmen" of the "Bell of Cooke" type. This is the only way in which the medical profession can make itself felt; and we are glad to see that the Austin District Medical Society will take the initiative in an aggressive campaign. Let every county and town society in the State follow their example, and *go to work* with their patients. It is not difficult to make an intelligent man see the point; and if every member of the State Association, and of every local society in Texas, will make it his business to enlighten his patrons on the necessities of restricting the privilege of practicing medicine to those who are educated to it, he can secure their promises to support for the Legislature only the candidate who has the intelligence to comprehend the subject, and the honesty and openness to pledge himself to the desired measure. The time has come for action,—let us all strike together, and the days of the pretender in medicine are numbered in Texas.

